# Assessment of role of perioperative melatonin in prevention and treatment of postoperative delirium after hip arthroplasty under spinal anesthesia in the elderly

**DOI:** 10.4103/1658-354X.71132

**Published:** 2010

**Authors:** Sherif S. Sultan

**Affiliations:** *Assistant Professor of Anesthesia and Intensive Care, Faculty of Medicine, Ain Shams University, Cairo, Egypt, Consultant, Department of Anesthesia, Al-Hada Armed Forces Hospital, Taif, Kingdom of Saudi Arabia*

**Keywords:** *Postoperative delirium*, *melatonin*, *hip arthroplasty*, *spinal anesthesia*

## Abstract

**Context:**

Little is known about the relationship between sedative drugs used preoperatively and postoperative delirium. Melatonin is a drug used to sedate patients preoperatively and is hypothesized by recent works to have a curative effect on postoperative delirium.

**Aims:**

The incidence of postoperative delirium will be tested if affected by three different sedative drugs including melatonin.

**Settings and Design:**

Controlled randomized doubleblind study.

**Patients and Methods:**

Three-hundred patients aged>65 years scheduled for hip arthroplasty under spinal anesthesia were randomly distributed to one of the four groups. Group 1 (control) received nothing for sedation. Group 2 (melatonin) received 5 mg melatonin. Group 3 (midazolam) received 7.5 mg midazolam. Group 4 (clonidine) received 100 *μ*g clonidine. These medications were given orally at sleep time at night of operation and another dose 90 min before operative time. Patients who developed postoperative delirium received 5 mg of melatonin 9 pm for three successive days in a trial to treat delirium.

**Statistical Analysis Used:**

Statistical analysis was done using the SPSS Software (version 13).

**Results:**

Total of 222 patients completed the study. Percentage of postoperative delirium in the control group was 32.65% (16/49 patients). The melatonin group showed a statistically significant decrease in the percentage of postoperative delirium to 9.43% (5/53 patients). Melatonin was successful in treating 58.06% of patients suffered postoperative delirium (36/62 patients) with no difference between different groups.

**Conclusions:**

Postoperative delirium is affected with the drug used for preoperative sedation. Melatonin was successful in decreasing postoperative delirium when used preoperatively and in treating more than half of patients developed postoperative delirium when used for three postoperative nights.

## INTRODUCTION

Delirium, which is also known as an acute confusional state, is a syndrome characterized by disturbance in consciousness (i.e., reduced clarity of awareness of the environment), change in cognition including alteration in attention, disorganized thinking, disturbed psychomotor activity, and abnormal sleep-wake cycle.[1] Postoperative delirium in the elderly occurs in 10% to 61% of those aged 65 or older.[2] Orthopedic patients are more likely to experience delirium than those undergoing general surgery. Delirium develops in 44% to 55% of hip surgery patients versus 10% to 14% of general surgery patients.[3] Once delirium develops, it is associated with a 10% to 75% mortality rate, although death may be related more to advanced age and severity of illness than to delirium per se.[4]

Medications are the most common reversible cause of delirium. It is estimated that medications contribute to 22% to 39% of all cases of delirium.[5] A study involving older hospitalized adults found that the most likely primary cause of delirium in their study population was medication use.[6]

Melatonin (*N*-acetyl-5-methoxytryptamine) is a hormone produced in the brain by the pineal gland from the amino acid tryptophan. Synthetic melatonin supplements have been used for a variety of medical conditions, most notably for disorders related to sleep. It was proved successful when used as a premedication to decrease anxiety and sedate patients preoperatively with an excellent cognitive profile.[7] Moreover, it was hypothesized to have a curative effect on postoperative delirium.[8] In this study, we try to set a general incidence of postoperative delirium in patients undergoing hip arthroplasty under spinal anesthesia. Further, this incidence will be tested if affected by three different sedative drugs including melatonin. Patients who might develop postoperative delirium would receive postoperative melatonin in a trial of control symptoms.

## PATIENTS AND METHODS

Three hundred patients were enrolled in this study. After approval of the departmental committee, an informed consent was attained from all patients or their responsible relatives. ASA I-III patients aged 65 years or more scheduled for hip arthroplasty were included in the study. Exclusion criteria are listed in Table 1. At night of operation, patients have been seen and examined by an anesthesia resident. Premedication drugs included in the study were given to the ward nurse in a closed envelope prepared and randomized by the hospital pharmacy within which an enclosed leaflet with the dose, timing, and precautions of the drug was included. The resident anesthetist was blinded to the drug inside the closed envelope. Appreviated Mental Test (AMT)[9] was conducted to all patients at that time [Table 2]. This test has been recommended for routine assessment of cognitive function in the elderly by the Royal College of Physicians and the British Geriatric Society.[10] Patients with scores of <8 were excluded from the study.

**Table 1:** Exclusion criteria

History of alcohol abuse
Sensory impairment (blindness, deafness)
Dementia
Severe infections (especially respiratory, urinary)
Severe anemia (hematocrit<30%)
Intracranial events (stroke, bleeding, infection)
Fluid or electrolyte disturbances, including dehydration, hyponatremia, hypernatremia
Acute cardiac events: myocardial infarction, congestive heart failure exacerbation, arrhythmia
Acute pulmonary events: asthma or chronic obstructive pulmonary disease exacerbation, pulmonary embolism, hypoxemia, hypercarbia
Medications
Anticonvulsants, esp. phenytoin
Antidepressants, esp. the tertiary amine tricyclic agents: amitriptyline, imipramine, doxepin
Antihistamines, including diphenhydramine
Antiparkinsonian agents: levodopa-carbidopa, dopamine agonists, amantadine
Antipsychotics: esp. low-potency anticholinergic agents and atypical agents (clozapine)
Benzodiazepines, esp. long-acting, including diazepam, flurazepam, chlordiazepoxide
Benzodiazepines, ultra-short-acting, including triazolam, alprazolam

**Table 2:** Abbreviated mental test

Age Time (to nearest hour) Address for recall at end of test: e.g. 42 West Street. (Ask patient to repeat the address to ensure it has been heard correctly) Year Name of hospital Recognition of two persons (e.g. doctor, nurse) Date of birth Year of start of first world war (or any famous event) Name of monarch Count backwards from 20 to 1

Each point scores one

Patients were allocated to one of the four groups. Group 1 (control) received nothing as premedication. Group 2 (melatonin) received one capsule of 5 mg melatonin (Sigma Chemical, St. Louis, MO) at sleep time and another 5 mg 90 min before operative time. Group 3 (midazolam) received one tablet of 7.5 mg midazolam (Roche, Auckland, NZ) at sleep time and another 7.5 mg 90 min before operative time. Group 4 (clonidine) received one tablet of 100 μg clonidine (Boehringer Ingelheim, Berkshire, UK) at sleep time and another 100 μg 90 min before operative time.

On arrival to the preanesthesia room, patients’ blood pressure as well as sedation score according to sedation score [Table 3] were reported. Then the patient was left to the attending anesthetist (who was blinded to the drug used as premedication) for the standard monitored care. Spinal anesthesia was given to all patients according to the standard techniques. AMT was repeated in the same day of operation (POD-0) and in the three postoperative days (POD) followed (POD-1, POD-2 and POD-3). Patients proved to have scores<8 were considered to develop postoperative delirium. These patients received melatonin 5 mg at 9 p.m. and reassessed by AMT daily for 3 successive days. A psychiatrist was consulted for patients that did not respond to treatment with melatonin for further evaluation and treatment.

Statistical analysis was done using SPSS Software (version 13). Descriptive statistics were done (e.g., number, percent) and analytic statistics (e.g., Student’s *t*-test to compare two independent means). *P*-value < 0.05 was considered as statistically significant.

**Table 3:** Sedation score

0:	Alert
1:	Arouses to voice
2:	Arouses with gentle tactile stimulation
3:	Arouses with vigorous tactile stimulation
4:	Unarousable

## RESULTS

Three hundred patients were tried to be enrolled in the study. Seventy eight of them had an AMT score of<8. This reveals an incidence of 26% delirium preoperatively. The rest of patients (222) were enrolled in the study. Eleven patients were further excluded due to the need to induce general anesthesia for different causes and another 8 patients for postoperative ICU admission. The remaining 203 patient completed the study. Distributions of cases, demographic, and surgical data are listed in Table 4. Data were comparable between groups.

**Table 4:** Demographic and surgical data

	Control	Melatonin	Midazolam	Clonidine
Number of patients	49	53	50	51
Age (years)	72.3 (6.4)	70.4 (71)	69.9 (8.2)	71.5 (6.8)
Gender (M/F)	22/27	24/29	26/24	27/24
Weight (Kg)	84.4 (12.6)	79.7 (14.6)	88.2 (7.9)	78.5 (113)
Duration of surgery (min)	1197 (36.7)	126.8 (44.9)	110.7 (40.8)	133.8 (332)
Blood transfused (ml)	6554 (112.5)	703.4 (98.2)	588.8 (133.6)	684.8 (106.6)

Data were expressed as mean (SD)

Data collected on arrival to the preanesthesia room are listed in Table 5. The control group showed systolic hypertension compared with all other groups. The clonidine group showed a significantly lower reading of blood pressure weather systolic or diastolic compared with all other groups.

**Table 5:** Blood pressure and sedation score of different groups

	Control	Melatonin	Midazolam	Clonidine
SBP	162.7 (22.7)	147.8 (12.4)$	1451 (145)$	133.9 (14.6)$#@
DBP	93.3 (12.8)	92.8 (10.6)	89.4 (11.3)	79.6 (13.9)$#@
Sedation score	0	1.9 (0.6)$	2.1 (0.5)$	1.8 (1)$

$ Significant compared with the control group;

# Significant compared with the melatonin group;

@ Significant compared with the midazolam group;

Data expressed as mean (SD)

None of patients were sedated in the control group. All patients in the other three groups were sedated to a comparable limit.

Postoperatively, numbers of patients developed delirium are listed in Table 6. The melatonin group differed significantly compared with all groups, having less number of patients developed delirium. The total number of patients prescribed postoperative melatonin for treatment of delirium was 62 patients. Their distribution between groups is listed in Table 6. Treatment was successful in 36 (58.06%) with no significant group differences.

**Table 6:** Number of patients developed delirium in different postoperative days

	Control	Melatonin	Midazolam	Clonidine
POD-0	0	0	2	0
POD-1	4	1	8	5
POD-2	8	1	10	9
POD-3	4	3	2	5
Total (percentage)*	16 (32.65)	5 (9.43)$	22 (44)#	19 (37.25)#
Number of patients treated with melatonin (percentage)¶	9 (56.25)	2 (40)	14 (63.63)	11 (57.89)

* Percentage to number of patients in each group;

¶ Percentage to patients developed postoperative delirium;

$ Significant compared with the control group;

# Significant compared with the melatonin group,

Figures in parentheses are in percentage

## DISCUSSION

Postoperative delirium is an underestimated problem by health care providers. Elderly patients in particular are more susceptible to develop postoperative delirium. Incidence of postoperative delirium will likely increase because of an aging population and more elderly patients presenting for major surgery.

The etiology of postoperative delirium is still not well understood. Conclusions from previous studies have not always been in complete agreement. However, studies delirium in both medical and surgical patients. Among elderly surgical patients in particular, preexisting patient factors as well as intraoperative and postoperative causes are also involved. Some studies have also highlighted important pre-and intraoperative factors that have not been shown to increase delirium. Among the multitude of factors associated with delirium, preoperative patientrelated factors are not modifiable. This is the reason behind developing “preoperative” delirium and delirium in medical wards of hospitals.[11] In this study the incidence of preoperative delirium (i.e. delirium diagnosed after hospital admission and before operative management) was 26%. It is known that about 15% of elderly have delirium upon admission to the hospital.[4] It is not surprising that the incidence is higher in our study because the population chosen was patients scheduled for hip arthroplasty. This group of patients have higher incidence of delirium in comparison with other elder patients. Delirium, as stated above, develops in 44% to 55% of hip surgery patients versus 10% to 14% of general surgery patients.[3]

Preoperative medications related to anesthesia-like sedatives- received little attention in previous studies. In our study patients without preoperative sedation developed delirium in a percentage of 32.56% of patients. When sedated preoperatively with oral midazolam or clonidine the incidence raised to 44 and 37.25%, respectively. However, both changes were statistically insignificant (*P*=0.245 and 0.629, respectively). Only preoperative melatonin succeeded in decreasing the incidence of postoperative delirium to 9.43% (*P*=0.003).

It is difficult to explain why midazolam and clonidine had higher incidence of postoperative delirium in our study compared with control (however still statistically insignificant) while melatonin had a statistically significant lower incidence. Delirium is a syndrome characterized by disturbance in consciousness (i.e., reduced clarity of awareness of the environment), change in cognition including alteration in attention, disorganized thinking, disturbed psychomotor activity, and abnormal sleep-wake cycle.[1] In the view of this definition, a review of mechanism of actions and clinical effects of these drugs may make it easier to understand the clinical difference concerning postoperative delirium. Midazolam is simply an agonist of GABA receptors. The use of midazolam is associated with significant impairment of psychomotor skills. Antrograde amnesia is characteristic with midazolam.[12] Midazolam (and most benzodiazepines) induces marked attenuation of rapid eye movement (REM) sleep.[13] It is well known that suppression of any specific sleep stage is followed by rebound response when the inhibition is removed.[14] Subsequently, the augmented rebound in REM sleep occurs with marked hemodynamic responses.^15^] Postoperative sleep disturbances are pronounced. It may be an important factor in the development of postoperative cerebral dysfunction including postoperative delirium.[14] Clonidine, the α-2 adrenergic agonist acts centrally through locus ceruleus where it reduces sympathetic outflow. It impairs psychomotor functions.[16,17] It also decreases REM sleep postoperatively with possible changes listed above.[13] These changes in cerebral function in general and postoperative sleep disturbances in specific are not confined to general anesthesia and occur after regional anesthesia in the same rate.[18]

On the other hand, melatonin is naturally produced by the human body and plays an important role in regulation of the sleep-wake cycle.[19] It has an endogenous circadian rhythm of secretion induced by the suprachiasmatic nuclei of the hypothalamus. Elderly are more prone to degeneration of these nuclei. This lowers the baseline serum melatonin levels in the elderly individuals.[20] Moreover, surgery induces more lowering of serum melatonin.[21,22] This produces a state of sleep disorders and disruption of REM sleep postoperatively. Exogenous administration of melatonin is known to facilitate sleep onset and improves quality of sleep.[23] Clinically, it has no amnestic properties and it does not produce impairment of cognitive and psychomotor skills or tests of memory and visual sensitivity.[24]

The success of melatonin it treatment of postoperative delirium was tried before but on the scale of case reports.[8] In this study we tried it for a total of 62 patients with a success rate of about 58%. Restoration of normal sleep-wake cycle which happened with administration of melatonin may be a key in treating postoperative delirium. However, it could not treat all cases presented because delirium is multifactorial problem and not very well understood.

In conclusion, postoperative delirium is an underestimated complication after anesthesia and surgery by most of clinicians. We were confronted by high incidence of “preoperative” and postoperative delirium. Testing different preoperative sedatives proved the sharing of sedative drugs in the problem of delirium. Midazolam and clonidine produced insignificant higher incidence of delirium, while melatonin had a significant reduction of incidence of postoperative delirium. Melatonin was successful in treating more than half of the cases of delirium when administered postoperatively to patients with postoperative delirium.
